# Porcine deltacoronavirus enters ST cells by clathrin-mediated endocytosis and does not require Rab5, Rab7, or Rab11

**DOI:** 10.1128/spectrum.02553-23

**Published:** 2023-11-14

**Authors:** Shiqian Li, Dai Xiao, Luwen Zhang, Rui Chen, Daili Song, Yiping Wen, Rui Wu, Qin Zhao, Senyan Du, Qigui Yan, Sanjie Cao, Xiaobo Huang

**Affiliations:** 1 Research Center for Swine Diseases, College of Veterinary Medicine, Sichuan Agricultural University, Chengdu, China; 2 Sichuan Science-Observation Experimental Station for Veterinary Drugs and Veterinary Diagnostic Technology, Ministry of Agriculture, Chengdu, China; 3 National Animal Experiments Teaching Demonstration Center, Sichuan Agricultural University, Chengdu, China; University of Georgia, Athens, Georgia, USA

**Keywords:** porcine deltacoronavirus (PDCoV), endocytosis, clathrin, Rab proteins

## Abstract

Porcine deltacoronavirus (PDCoV) is a newly emerged enteric pathogen that causes diarrhea, dehydration, vomiting, and in suckling piglets, a high rate of death. Here, we used endocytosis inhibitors, dominant-negative mutants, and small interfering RNA (siRNA) interference to investigate PDCoV entry into swine testicle (ST) cells. Results showed that chlorpromazine, dynasore, and methyl-β-cyclodextrin (MβCD) inhibited PDCoV internalization; immunofluorescence assay (IFA) of dominant-negative (DN) mutants and siRNA interference further demonstrated these results, indicating that PDCoV utilizes clathrin-mediated endocytosis and requires dynamin and cholesterol to enter ST cells. The role of endosomes in PDCoV infection was also investigated and we found that PDCoV does not require Rab5, Rab7, or Rab11 for productive infection, indicating that the transport of PDCoV in ST cells does not require the participation of the endosomal system. Our findings will provide additional understanding of the entry mechanisms of PDCoV and possible antiviral targets.

**IMPORTANCE:**

Porcine deltacoronavirus (PDCoV) is a newly emerged enteric virus threatening pig industries worldwide. Our previous work showed that PDCoV enters porcine kidney (PK-15) cells through a caveolae-dependent pathway, but the entry mechanism for PDCoV into swine testicle (ST) cells remains unclear. Mechanisms of virus entry can be different with different virus isolates and cell types. Here, we determined that PDCoV enters ST cells via clathrin-mediated endocytosis. Additionally, we found that PDCoV entry does not require Rab5, Rab7, or Rab11. These findings provide additional understanding of the entry mechanisms of PDCoV and possible antiviral targets.

## INTRODUCTION

Porcine deltacoronavirus (PDCoV) (genus *Deltacoronavirus*, family *Coronaviridae*) is a porcine enterovirus that clinically manifests as diarrhea, dehydration, and vomiting; in neonatal piglets, the mortality rate is nearly 100% (1). Pathologically, in addition to extensive intestinal lesions, PDCoV also causes significant gastric lesions and mild lung lesions (2). Outbreaks of PDCoV were first reported in the United States in 2014, and subsequently have been reported in Canada, China, Korea, Laos, and numerous other countries (3 – 7).

While epidemiological investigations have indicated that both gamma- and deltacoronavirus originated from avian coronavirus (8), PDCoV can set up infection in artificially inoculated calves, chickens, and turkeys (9, 10), and to a limited extent, in mice (11). Recently, three cases of PDCoV infection were reported in Haitian children with acute undifferentiated febrile illness (12). These findings indicate the potential for wide cross-species transmission and a growing public health risk.

The PDCoV genome is approximately 25 kb and has seven open reading frames, [1a/1b (ORF1a/1b), spike (S), envelope (E), membrane (M), nucleocapsid (N), NS6, and NS7]. To date, knowledge of the pathogenic mechanism of PDCoV and of the receptors for entry into host cells is incomplete. Li et al. (13) found that aminopeptidase N (APN) is a functional receptor for PDCoV in a variety of animal cells, but other studies indicate that APN is a non-essential receptor for PDCoV (14), and that it is a co-factor for PDCoV entry via the endocytic pathway (15). Yang et al. (16) report that trypsin promotes PDCoV replication by mediating cell-to-cell fusion, but it is not crucial for viral entry. In 2018, Jeon and Lee (17) found that cholesterol present in viral capsule and host cell membranes affects PDCoV entry. Later, Zhang et al. (18) found that PDCoV enters cells through both the endocytic pathway and the cell membrane surface pathway. Porcine deltacoronavirus enters porcine IPI-2I intestinal epithelial cells via macropinocytosis and clathrin-mediated endocytosis dependent on pH and dynamin (19). Our previous work also found that PDCoV enters PK-15 cells through caveolae-mediated endocytosis (CavME) (20).

Viruses can enter cells through several endocytic pathways: clathrin-mediated endocytosis, caveolae/lipid raft-mediated endocytosis, macropinocytosis, and clathrin/caveolae-independent endocytosis. Human coronavirus (HCoV-NL63), CoVID-19, mouse hepatitis virus (MHV-2), avian infectious bronchitis virus (IBV), and porcine hemagglutinating encephalomyelitis virus (PHEV) enter cells through clathrin-mediated endocytosis (21 – 25); HCoV-229E, HCoV-OC43, and canine respiratory coronavirus (CRCoV) enter cells through caveolae-mediated endocytosis (26 – 28); ebolavirus and African swine fever virus (ASFV) enter cells through macropinocytosis (29, 30); and feline infectious peritonitis viruses (FIPV) and SARS-CoV enter via clathrin/caveolae-independent endocytosis (31, 32). In addition, some viruses can use multiple endocytic pathways to enter the same cell, for example, transmissible gastroenteritis virus (TGEV) can enter swine testicle (ST) cells through clathrin- and caveolin-mediated endocytosis (33).

Endocytic transport is regulated by a series of Rab small GTPases. Rab small GTPases are involved in vesicle transport and about three-quarters of them have a role in endocytic transport (34). Each Rab family protein is located on specific membranous organelles. For example, Rab5 is located on early endosomes and is the main regulator of early endocytic transport (35). Rab7 controls the maturation of early endosomes, the transport from late endosomes to lysosomes, the biogenesis of lysosomes, the aggregation and fusion of late endosomes and lysosomes in the perinuclear region, and the pH of late endosomes and lysosomes (36, 37). Rab11 regulates the endosomal recycling pathway (38).

Successful entry into cells is a major determinant of successful viral replication (15). Currently, our understanding of how PDCoV enters various cells is not complete. The cells typically used to study PDCoV include LLC-PK1, ST, PK-15, IPI-2I, and IPEC-J2. The endocytosis of PDCoV into PK-15 and IPI-2I cells has been well studied, while endocytosis into ST cells has not. Here, we used endocytosis inhibitors, dominant-negative expressing plasmids, and siRNA interference to study the entry mechanism of PDCoV into ST cells.

## MATERIALS AND METHODS

### Virus, plasmids, and cells

The PDCoV strain CHN-SC2015 (GenBank accession no. MK355396) (1) was provided by the Research Centre for Swine Disease of Sichuan Agricultural University. The expression plasmids of wild-type and dominant-negative EPS15 (DIII∆2 and E∆95/295), dynamin II (GFP-Dyn-WT and GFP-Dyn-DN), caveolin-1 (GFP-Cav-WT and GFP-Cav-DN), WT and DN Rab5 (S34N), WT and DN Rab7 (T22N), and WT and DN Rab11 (S25N) were kindly provided by Prof. Zhou Bin, Nanjing Agricultural University (Nanjing, China). ST cells were maintained at 37°C in a humidified 5% CO_2_ atmosphere in Dulbecco’s modified Eagle medium (DMEM; Gibco, Carlsbad, CA, USA) supplemented with 10% heat-inactivated fetal bovine serum (NEWZERUM, New Zealand) and 1% antibiotic-antimycotic (Solarbio, Beijing, China).

### Reagents and antibodies

The endocytic inhibitors dynasore, chlorpromazine (CPZ), methyl-β-cyclodextrin (MβCD), nystatin, ammonium chloride (NH_4_Cl), and 5-(N-ethyl-N-isopropyl) amiloride (EIPA) were purchased from Selleck Chemicals (Houston, TX, USA). Anti-β-actin rabbit mAb (dilution 1:10,000), anti-clathrin heavy-chain (CLTC) rabbit polyclonal (dilution 1:1,000), and anti-Rab5, Rab7, Rab11 rabbit polyclonal (dilution 1:1,000) coupled with secondary goat anti-rabbit IgG antibodies were purchased from ABclonal Technology (Wuhan, China). Alexa Fluor 555 donkey anti-rabbit IgG (H + L) secondary antibody was purchased from Beyotime Biotechnology (Shanghai, China). The rabbit anti-PDCoV N polyclonal antibody was generated by our laboratory (dilution 1:1,000).

### Cell viability and drug treatment

Cell viability was assayed as described previously (20). Briefly, ST cells were seeded into 96-well plates and treated with different concentrations of inhibitors (100 μL) for 2 h at 37°C; after two washes with phosphate buffer solution (PBS), 10 µL of cell counting kit-8 (CCK-8) solution was added to 90 µL of DMEM in each well and incubated for 1 h at 37°C. Absorbance at 450 nm was measured using a microplate reader. After determining the optimal working concentration, confluent monolayers of ST cells in six-well plates were treated with inhibitor for 1 h at 37°C, then inoculated with PDCoV (MOI = 5) and incubated for 1 h at 4°C (binding step) then shifted to 37°C. At 24 hpi, supernatant and cells were collected for subsequent assays.

### TCID_50_ assay

ST cells were seeded into 96-well plates and cultured until 90% confluent. Cell monolayers were washed twice with maintenance medium containing 5 µg/mL trypsin and then inoculated with 100 µL of 10-fold serial dilutions of infected cell supernatant; there were eight replicates per dilution. After incubation for 1.5 h at 37°C, 150 µL of maintenance medium was added to each well. Viral cytopathic effect (CPE) was observed over 4–6 days, and titers were calculated using the Reed and Muench method and expressed as 50% tissue culture infectious dose (TCID_50_) per milliliter.

### RNA extraction and quantitative real-time PCR

Total RNA was extracted using a UNlQ-10 Column TRIzol Total RNA Isolation Kit (Sangon, Shanghai, China), and then subjected to qRT-PCR using primers specific to the *N* gene of PDCoV. Primer sequences are listed in Table 1. ChamQ Universal SYBR qPCR Master Mix (Vazyme, Nanjing, China) was used for the qRT-PCR reactions, and the amplification conditions were 95°C for 30 s, then 40 cycles of 95°C for 10 s, 60°C for 30 s, and 72°C for 30 s using the LightCycler 96 system (Roche, Mannheim, Germany). The mRNA relative expression of PDCoV *N* was calculated using the 2^−ΔΔCt^ method with β-actin as internal reference.

**TABLE 1:** Primers for qRT-PCR

Primer	Sequence (5′→3′)
PDCoV N-F	CTATGAGCCACCCACCAA
PDCoV N-R	TCCCACTCCCAATCCTGT
β-Actin-F	CTTCCTGGGCATGGAGTCC
β-Actin-R	GGCGCGATGATCTTGATCTTC
CAV1-F	GATCCCAAGCATCTCAACGA
CAV1-R	AGAGGGCAGACAGCAAACG
DNM2-F	TCCCACTCGCAAGACCAAA
DNM2-R	CTCAAAGGGAAACCGCTCA

### Western blotting

The cells were washed with PBS and then incubated on ice in RIPA lysis buffer containing phenylmethanesulfonyl fluoride (PMSF) for 20 min. Boiled at 95°C for 10 min with loading buffer. Take out 20 μg total protein for SDS-PAGE, 80 V, 25 min; 120 V, 90 min. After separation of SDS-PAGE, proteins were transferred onto polyvinylidene fluoride (PVDF) membranes. The membranes were blocked with 5% skim milk in PBST (PBS/0.05% Tween-20) for 1.5 h and then incubated with the appropriate antibodies overnight at 4°C. Membranes were washed four times with PBST and then incubated with HRP-goat anti-rabbit IgG (1:5,000) for 1 h at 37°C. Membranes were again washed four times with PBST, and the proteins were visualized using enhanced chemiluminescence reagents (Bio-Rad, Hercules, CA, USA).

### Transfection and immunofluorescence

ST cells were grown to 50% confluence on coverslips and transfected with 2.5 µg of the indicated plasmids using Lipofectamine 3000, according to the manufacturer’s instructions. After 24 h, the cells were infected with PDCoV (MOI = 0.1). At 6 hpi, cells were fixed in 4% paraformaldehyde for 30 min, permeabilized with 0.2% Triton X-100 for 30 min, blocked with 5% bovine serum albumin for 1 h at 37°C, then incubated overnight with rabbit anti-PDCoV N polyclonal antibody (1:200) at 4°C. Membranes were washed again and incubated with Alexa Fluor 555-labeled donkey anti-rabbit IgG (H + L) (1:500) for 1 h at room temperature. Cell nuclei were stained with DAPI, and cells were observed using a Pannoramic Desk slide scanner (3DHISTECH, Hungary).

### Plasmid construction and lentivirus production

In order to interfere with the expression of endocytosis-related proteins, we used CRISPR/Cas9 technology and lentivirus packaging technology to construct knockdown cell lines. The methods used are described in Li et al. (20). Briefly, we used the website http://chopchop.cbu.uib.no/ to design sgRNAs and their complementary chains to construct plasmids targeting the *Sus scrofa* genes: *CLTC*, caveolin-1 (*CAV1*), dynamin-2 (*DNM2*), *Rab7*, and *Rab11*. The sgRNA sequences (listed in Table 2) were cloned into pLenti-CMV-V2-puro. The recombinant plasmids along with packaging plasmid (psPAX2) and envelope plasmid (pMD2.G) were co-transfected into HEK293T cells using Lipofectamine 3000. At 36 and 48 h post-transfection, supernatants (containing lentivirus) were collected. ST cells were inoculated with the lentiviruses and incubated for 36 h, the overlay medium was then changed to fresh DMEM containing 1.5 µg/mL puromycin. Stable cell lines were isolated from these cultures.

**TABLE 2:** sgRNA target sequences

Gene	Sequence (5′→3′)
CLTC	ATGCGTATCAGTCCAGATCA
CAV1	AGTGTATGACGCGCACACCA
DNM2	GTTCAGGAATCGTCACTCGG
Rab7	ACGGTTCCAGTCTCTTGGCG
Rab11	GGTAGTCGTACTCGTCGTCG

### Rab proteins in PDCoV infection

To investigate the relationship between PDCoV infection and expression of Rab5, Rab7, and Rab11, we incubated ST cells with PDCoV (MOI = 0.1) for 24 h at 37°C. Samples were collected at 0, 6, 12, 18, and 24 hpi for Western blotting analysis.

### Statistical analysis

All data are presented as the means ± standard deviations (SDs) from at least three independent experiments. Significance was estimated using two-tailed Student’s *t*-tests. *P* values <0.05 were defined as the threshold for statistical significance. *P* values between 0.05 and 0.01 are designated by one asterisk, *P* values between 0.01 and 0.001 are designated by two asterisks, *P* values between 0.001 and 0.0001 are designated by three asterisks, and *P* values <0.0001 are designated by four asterisks. All graphs were created using GraphPad Prism 8 software.

## RESULTS

### Cell viability

**Fig 1:**
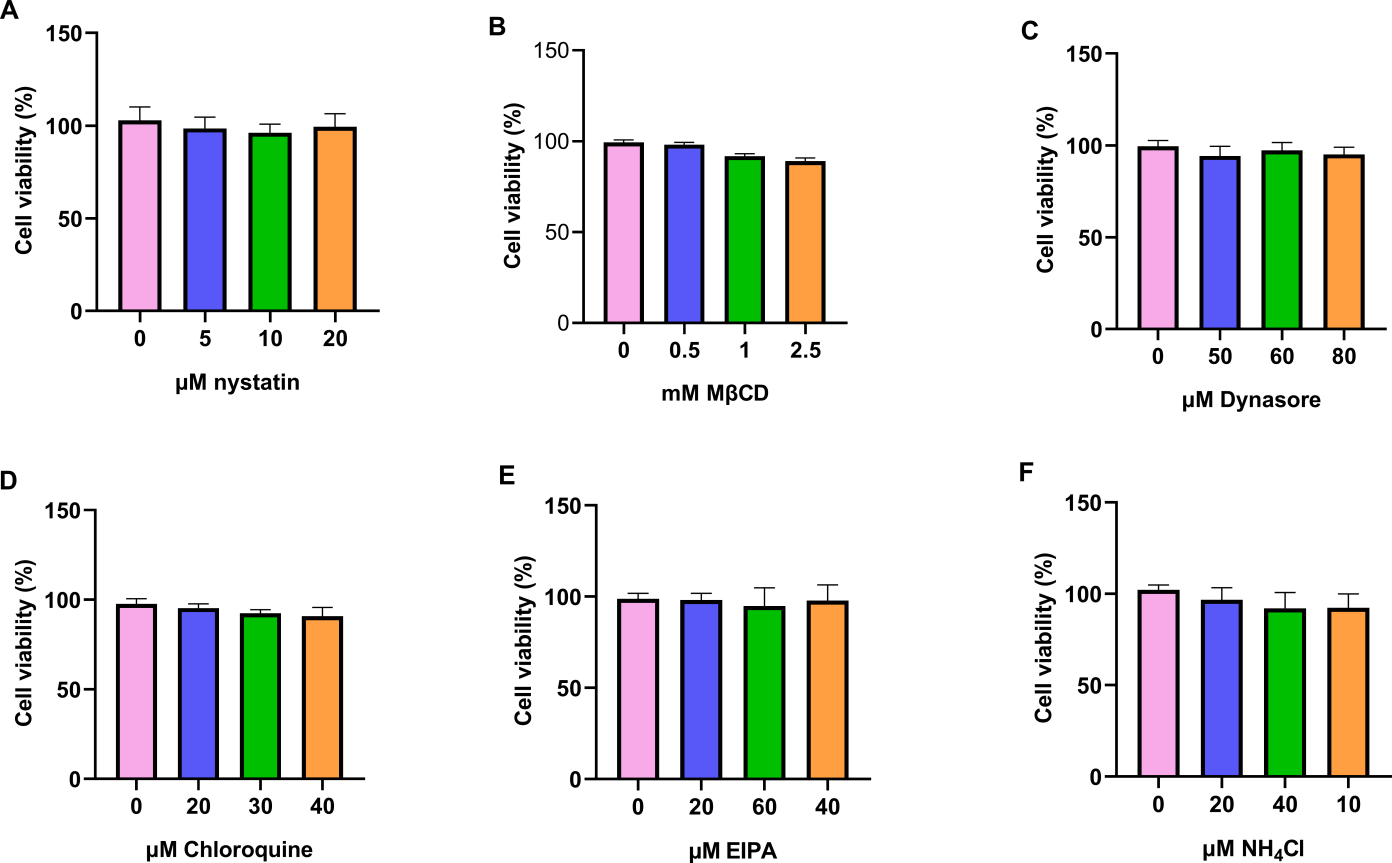
Cell viability. ST cells were seeded into 96-well plates at 3 × 10^4^ cells/well, incubated for 24 h and treated with inhibitors or left untreated for 2 h. After two washes with PBS, 10 µL of CCK-8 solution was added to each well and incubated for 1 h at 37°C. Absorbance was measured at 450 nm. The bars indicate the mean ±  SD from three independent experiments. (A) Nystatin, (B) MβCD, (C) dynasore, (D) chloroquine, (E) EIPA, and (F) NH_4_Cl.

The results of the cytotoxic assays are illustrated in Fig. 1. At the highest concentration of each inhibitor tested (nystatin: 20 µM; MβCD: 2.5 mM; dynasore: 80 µM; chlorpromazine: 40 µM; EIPA: 60 µM; NH_4_Cl: 40 mM), cell viability was greater than 90%.

### PDCoV utilizes on clathrin-mediated endocytosis for entry into ST cells

**Fig 2:**
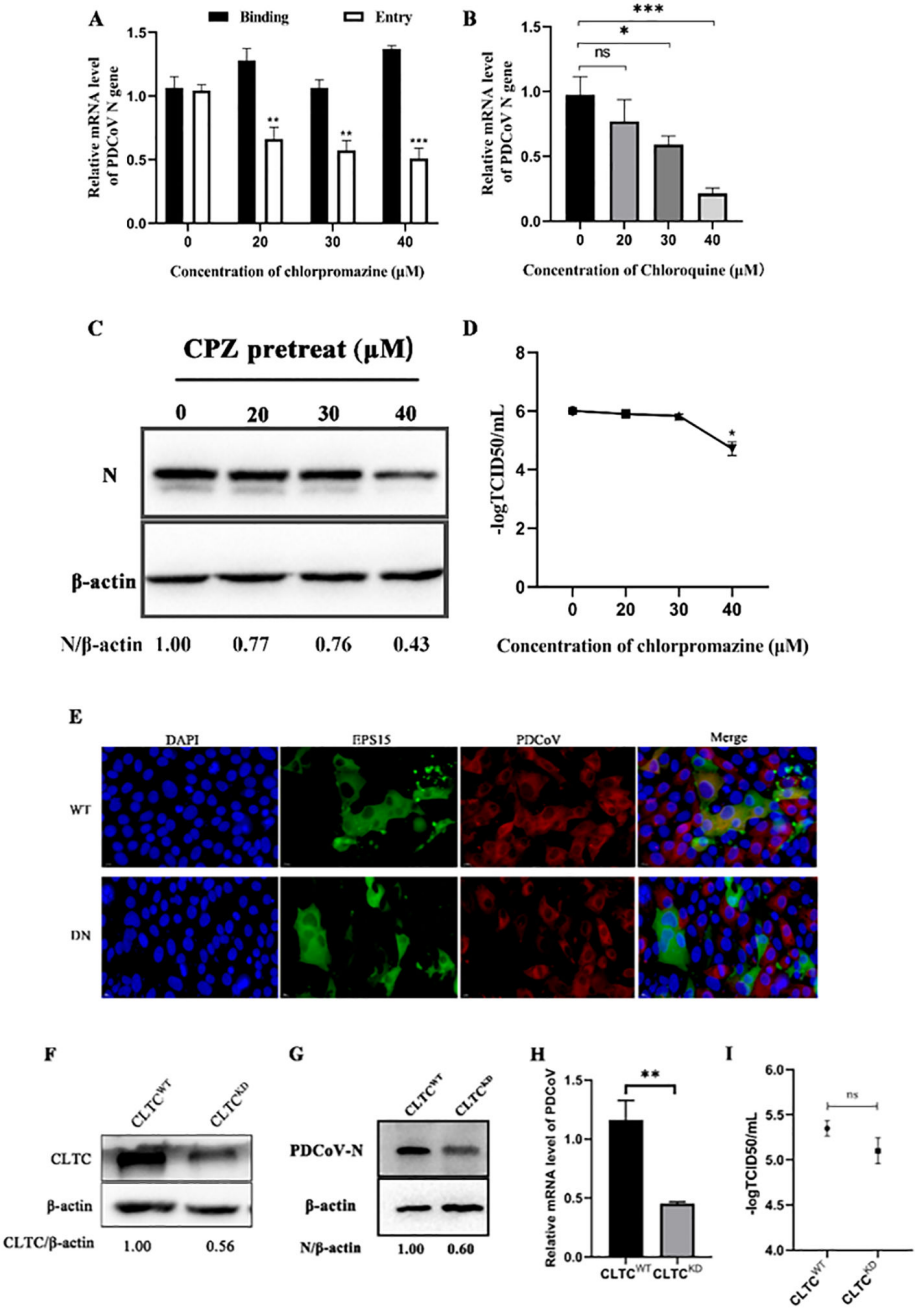
PDCoV entry into ST cells depends on clathrin-mediated endocytosis. (A) ST cells were treated with CPZ for 1 h at 37°C, then incubated with PDCoV (MOI = 5) for 1 h at 4°C (binding step), and then shifted to 37°C for 1 h (entry step). qRT-PCR of cell lysates was done to determine viral mRNA levels. (B–D) Cells were mock-treated or treated with CPZ for 1 h followed by infection with PDCoV (MOI = 0.1). At 24 hpi, cell lysates were used for (B) qRT-PCR to determine viral mRNA levels, (C) Western blotting to determine intracellular N protein levels, and (D) TCID_50_. (E) ST cells were transfected plasmids expressing with WT or DN EPS15 for 24 h, followed by infection with PDCoV (MOI = 0.1) for 6 h, then fixed and immuno-stained. The magnification is 80×. (F–I) Clathrin knockdown and normal cells were infected with PDCoV (MOI = 0.1) for 24 h. (F) The knockdown efficiency was determined by Western blotting. PDCoV (G) N protein levels, (H) TCID_50_, and (I) *N* mRNA levels were determined. *P < 0.05, **P < 0.01, ***P < 0.001, and ns: no significant difference.

To determine whether PDCoV enters ST cells via clathrin-mediated endocytosis, we started by infecting cells in the presence of CPZ. CPZ inhibits the assembly of coated pits by blocking the formation of clathrin and AP2 complexes on the cytoplasmic membrane. As can be seen from Fig. 2A, CPZ had no effect on the binding of virus but significantly inhibited PDCoV entry. By qRT-PCR and Western blot, we found that PDCoV infection decreased with increasing CPZ (Fig. 2B through D). At 20, 30, and 40 µM, the mRNA levels of PDCoV *N* decreased 24%, 41%, and 78%, respectively, and N protein levels decreased by 23%, 24%, and 57%, respectively. However, the titer of PDCoV was significantly decreased only at 40 µM (Fig. 2D). These results indicate that PDCoV enters ST cells using the CME pathway.

Wild-type and dominant-negative EPS15 expressing plasmids were transfected into ST cells followed by PDCoV infection. By indirect immunofluorescence, we found EPS15 and PDCoV colocalized in WT^+^ but not DN^+^ cells (Fig. 2E). These results further indicate that PDCoV enters ST cells via the CME pathway.

To further substantiate these results, expression of CLTC was knocked down in ST cells; knockdown efficiency was determined by Western blotting (Fig. 2F). CLTC knockdown and normal cells were infected with PDCoV for 24 h, then cells and supernatants were collected for Western blotting, qRT-PCR, and TCID_50_ (Fig. 2G through I). Compared with normal cells, the N protein levels and mRNA levels were significantly lower in KD cells (40% and 60%, respectively), but the viral titer was not. These results demonstrate that downregulation of clathrin inhibits PDCoV entry, further demonstrating that PDCoV utilizes the CME pathway for entry into ST cells.

### PDCoV entry is caveolae independent

**Fig 3:**
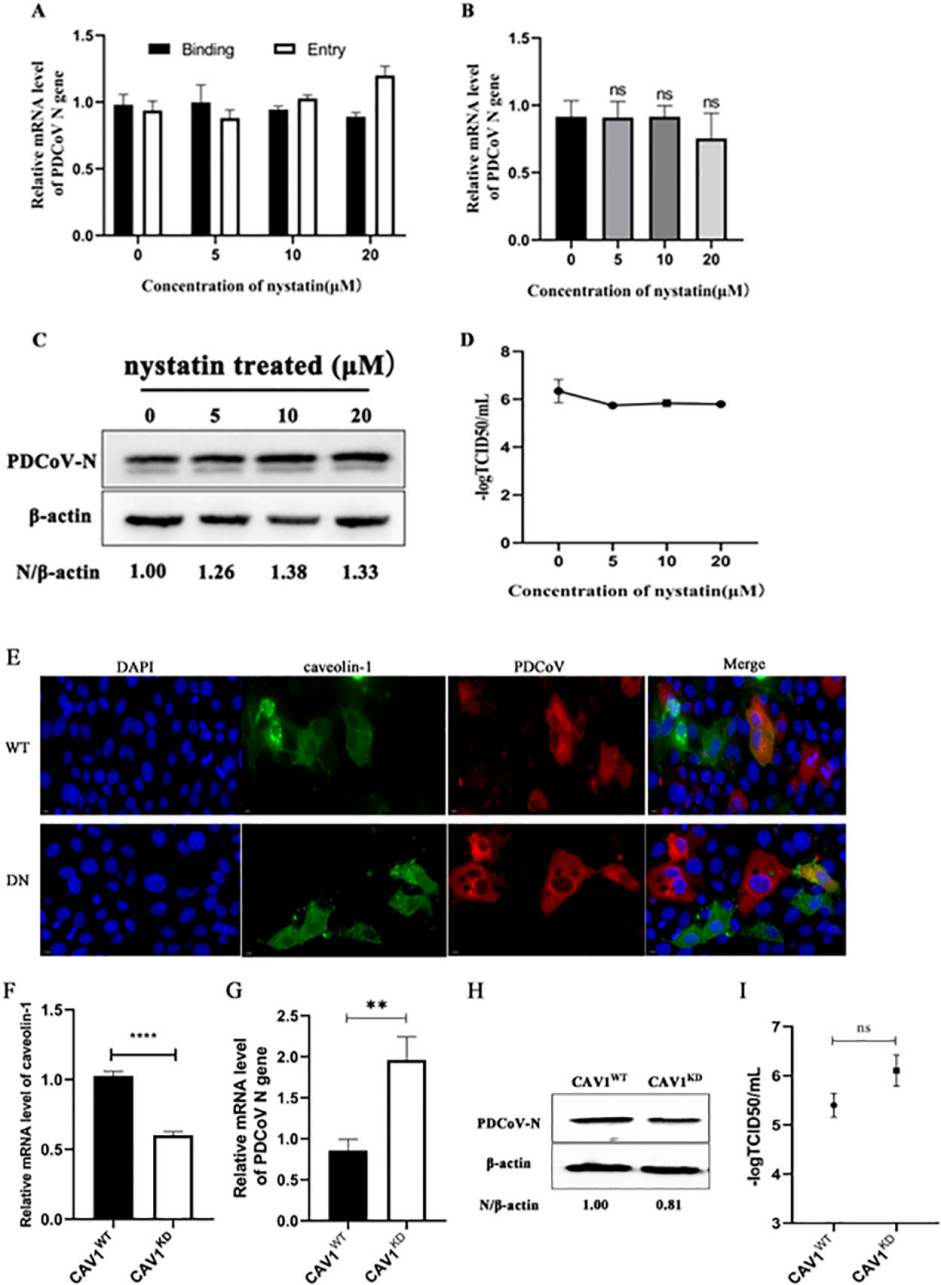
PDCoV entry is caveola independent. (A) ST cells were pretreated with nystatin for 1 h at 37°C, then incubated with PDCoV (MOI = 5) for 1 h at 4°C (binding step), and then shifted to 37°C for 1 h (entry step). Viral *N* mRNA was quantitated by qRT-PCR. (B–D) Cells were mock-treated or treated with nystatin for 1 h followed by infection with PDCoV (MOI = 0.1) for 24 h. (B) *N* mRNA levels were quantitated by qRT-PCR. (C) Intracellular N protein levels were detected by Western blot. (D) Viral titers were determined by TCID_50_. (E) ST cells were transfected with caveolin-1 WT or DN expressing plasmids for 24 h, followed by PDCoV infection (MOI = 0.1) for 6 h. Cells were fixed and immuno-stained. The magnification is 80×. (F–I) Caveolin-1 knockdown and normal cells were infected with PDCoV (MOI = 0.1). (F) Knockdown efficiency was determined by qRT-PCR. At 24 hpi, (G) *N* mRNA levels, (H) viral titers, and (I) N protein levels were determined. *P < 0.05, **P < 0.01, ***P < 0.001, and ns: no significant difference.

Previous work has shown that PDCoV enters PK-15 cells by CavME (20). Here, we examined PDCoV entry into ST cells using nystatin, a sterol binder that breaks down caveolae. Binding and entry assay were performed. The results showed that nystatin had no significant effect on PDCoV binding and entry (Fig. 3A), nor on PDCoV *N* mRNA or protein levels, or titer (Fig. 3B through D).

Next, plasmids expressing WT and DN caveolin-1 were transfected into ST cells, followed by infection with PDCoV. By indirect immunofluorescence, we found caveolin-1 and PDCoV colocalized in WT^+^ cells and DN^+^ cells (Fig. 3E). The role of caveolin during PDCoV entry was further assessed by knockdown of caveolin-1; the knockdown efficiency was quantitated by qRT-PCR (Fig. 3F). Caveolin-1 knockdown cells infected with PDCoV had significantly greater *N* mRNA and protein levels than in WT cells, but there was no significant difference in virus titer (Fig. 3G through I). Taken together, these results indicate that PDCoV does not depend on the CavME pathway for entry into ST cells.

### PDCoV entry into ST cells is independent of macropinocytosis and low pH

**Fig 4:**
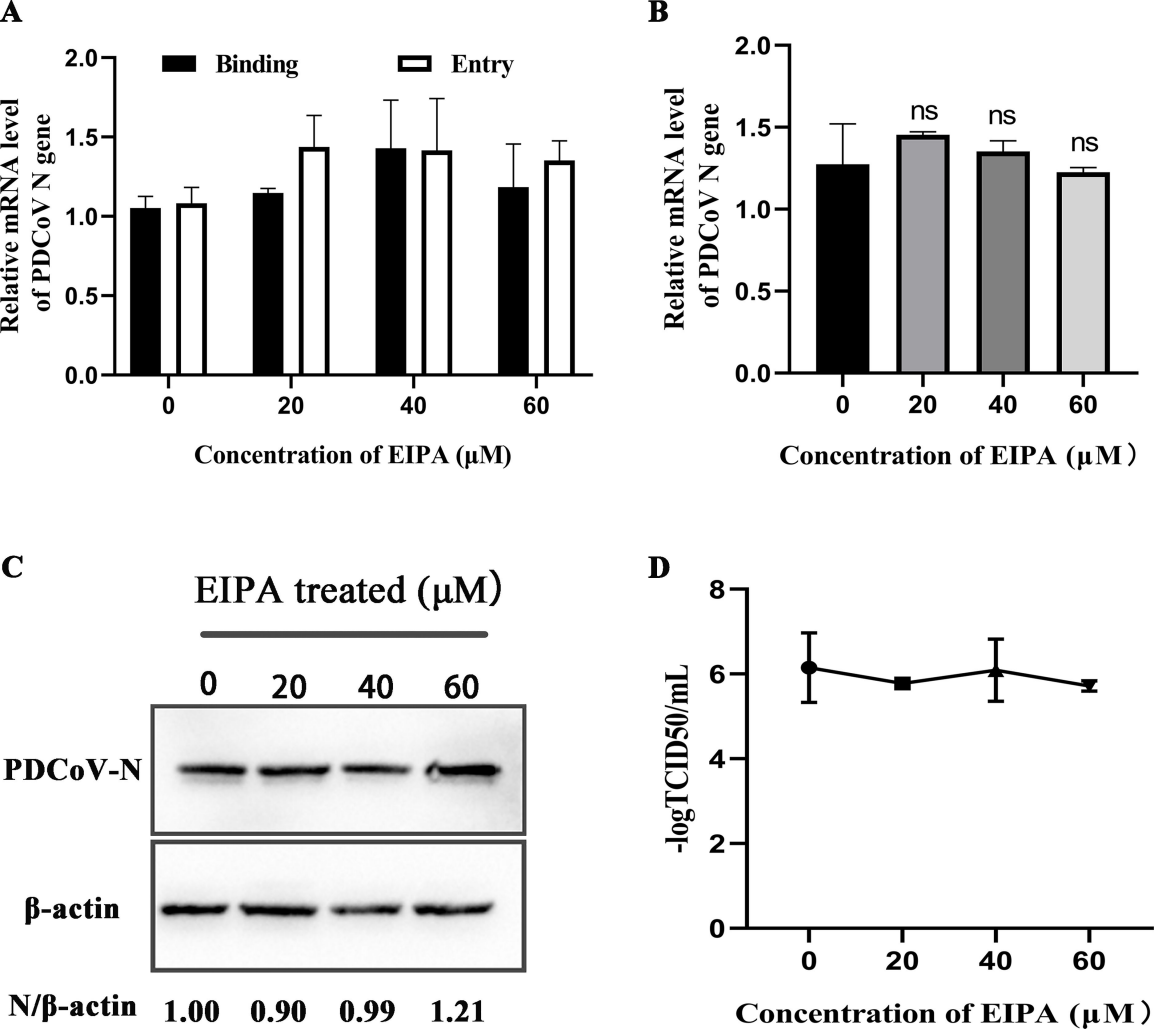
PDCoV entry into ST cells is independent of macropinocytosis. (A) ST cells were pretreated with EIPA for 1 h at 37°C, then incubated with PDCoV (MOI = 5) for 1 h at 4°C (binding step), and then shifted to 37°C for 1 h (entry step). Viral *N* mRNA was quantitated by qRT-PCR. (B–D) Cells were mock-treated or treated with EIPA for 1 h followed by infection with PDCoV (MOI = 0.1) for 24 h. (B) *N* mRNA levels were quantitated by qRT-PCR. (C) Intracellular N protein levels were detected by Western blot. (D) Viral titers were determined by TCID_50_. ns: no significant difference.

To evaluate the role of macropinocytosis in PDCoV entry, we examined the effect EIPA on infection. EIPA inhibits the macropinocytosis by blocking Na^+^/H^+^ exchange. The results of binding and entry assay showed that EIPA had no significant effect on PDCoV binding and entry (Fig. 4A), nor on PDCoV *N* mRNA or protein levels, or titer (Fig. 4B through D). These results indicated that PDCoV entry into ST cells does not depend on the macropinocytosis pathway.

**Fig 5:**
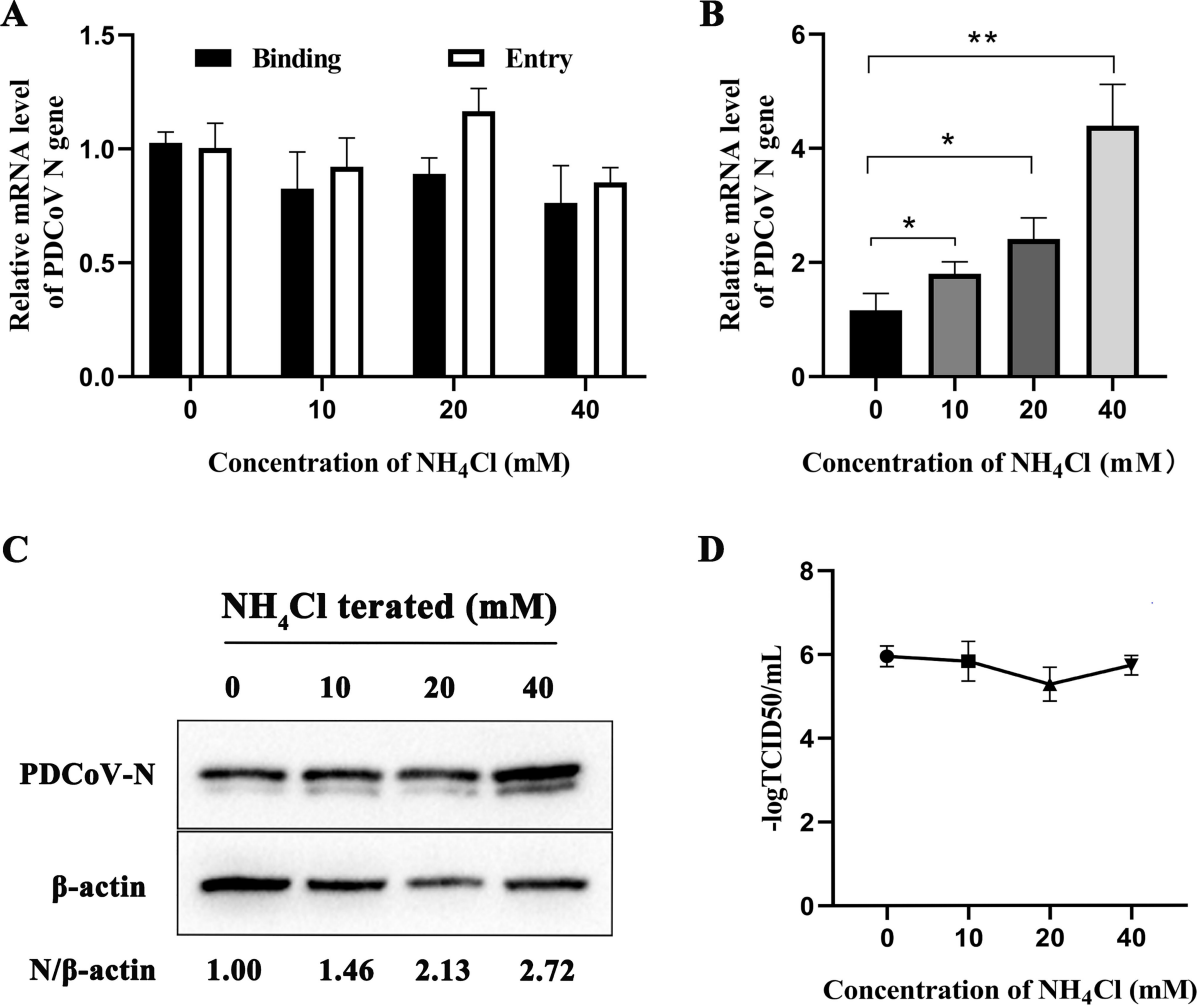
PDCoV entry into ST cells does not require low pH. (A) ST cells were pretreated with NH_4_Cl at 37°C for 1 h and infected with PDCoV (MOI = 5) at 4°C for 1 h (binding step), and then shifted to 37°C for 1 h (entry step). Viral *N* mRNA was quantitated by qRT-PCR. (B–D) Cells were mock-treated or treated with NH_4_Cl for 1 h followed by infection with PDCoV (MOI = 0.1) for 24 h. (B) *N* mRNA levels were quantitated qRT-PCR. (C) Intracellular N protein levels were detected by Western blot. (D) Viral titers were determined by TCID_50_. *P < 0.05, **P < 0.01, and ***P < 0.001.

Endosomal transport is regulated by intracellular pH and a series of small G proteins. To determine whether PDCoV entry into ST cells requires low pH, ST cells were treated with NH_4_Cl, an inhibitor of acidification. Results showed that NH_4_Cl had no significant influence on PDCoV cell binding or entry (Fig. 5A). *N* mRNA and protein levels did increase significantly with increasing concentration of EIPA but viral titers were not affected (Fig. 5B through D). These results indicate that PDCoV entry into ST cells does not depend on low pH.

### PDCoV entry into ST cells is dynamin dependent and requires cholesterol

**Fig 6:**
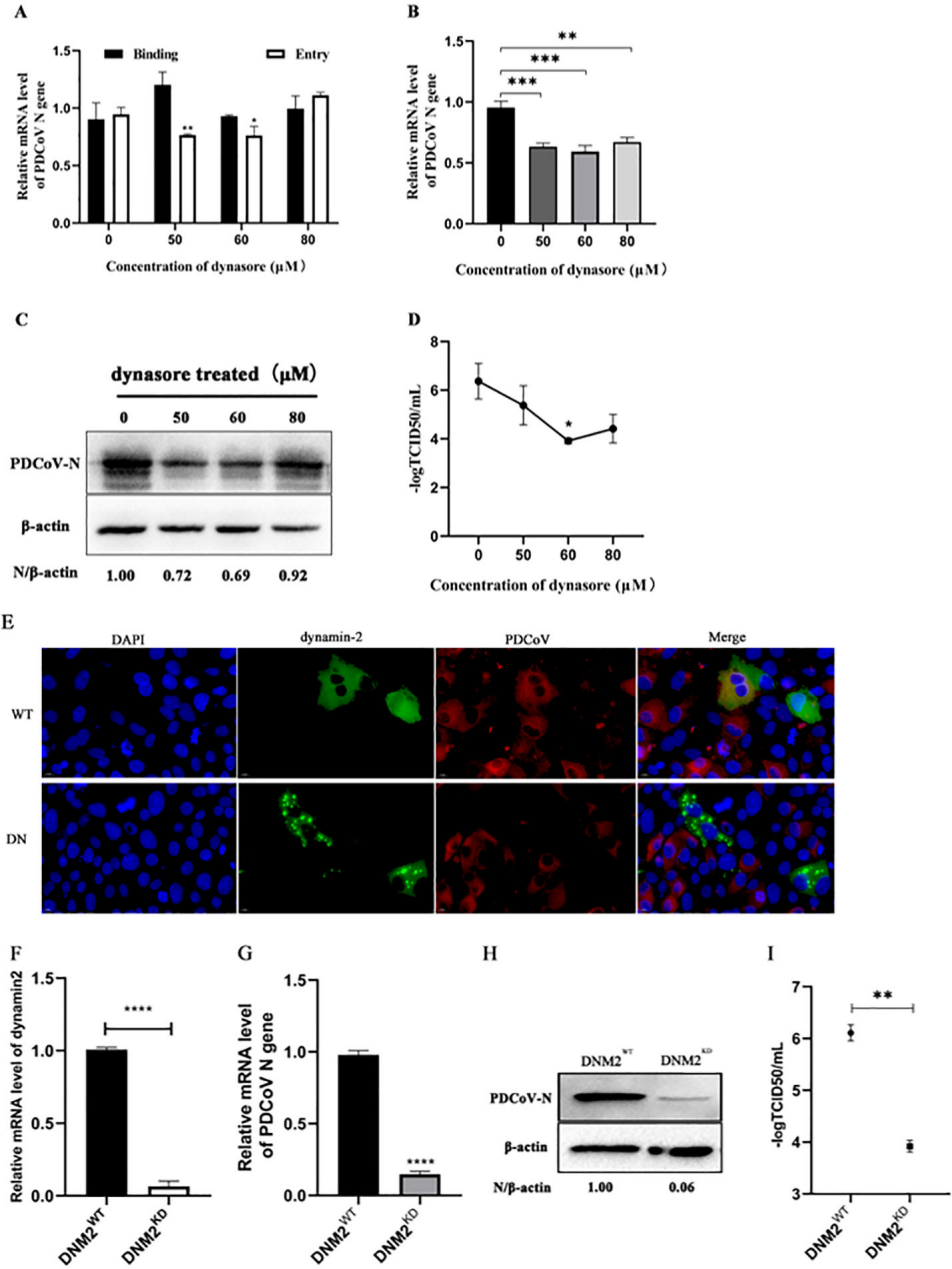
PDCoV entry into ST cells is dynamin dependent. (A) ST cells were pretreated with dynasore for 1 h at 37°C, then incubated with PDCoV (MOI = 5) for 1 h at 4°C (binding step), and then shifted to 37°C for 1 h (entry step). Viral *N* mRNA was quantitated by qRT-PCR. (B–D) Cells were mock-treated or treated with dynasore for 1 h followed by infection with PDCoV (MOI = 0.1) for 24 h. (B) *N* mRNA levels were quantitated by qRT-PCR. (C) Intracellular N protein levels were detected by Western blot. (D) Viral titers were determined by TCID_50_. (E) ST cells were transfected with dynamin-2 WT or DN expressing plasmids for 24 h, followed by infection with PDCoV (MOI = 0.1) for 6 h. Cells were fixed and immuno-stained. The magnification is 80×. (F–I) Dynamin-2 knockdown and normal cells were infected with PDCoV (MOI = 0.1). The knockdown efficiency was verified by qRT-PCR (F). At 24 hpi, the mRNA level of PDCoV, virus titer, and N protein level were determined by qRT-PCR (G), TCID50 (H), and Western blotting (I), respectively. *P < 0.05, **P < 0.01, ***P < 0.001, and ****P < 0.0001.

Clathrin and caveolin coated vesicles require dynamin to separate from the plasma membrane. Therefore, to determine whether dynamin is involved in PDCoV entry and infection, we first treated cells with the dynamin inhibitor dynasore. Results showed that dynasore had no significant effect on PDCoV binding, but significantly inhibited PDCoV entry at concentrations of 50 and 60 µM, although not at 80 µM (Fig. 6A). As shown in Fig. 6B through D, *N* mRNA and protein levels, as well as viral titers, decreased with increasing dynasore concentration up to 60 µM. At 80 µM, these levels had increased again. These results indicated that dynamin may be involved in PDCoV entry into ST cells.

Next, plasmids expressing WT and DN dynamin-2 were transfected into ST cells followed by infection with PDCoV. By indirect immunofluorescence (Fig. 6E), we found dynamin-2 and PDCoV colocalized in WT^+^ cells only, indicating that cells transfected with DN dynamin-2 could not be infected with PDCoV. The role of dynamin during PDCoV entry was further assessed by knockdown of dynamin-2; the knockdown efficiency was quantitated by qRT-PCR (Fig. 6F). Dynamin-2 knockdown cells infected with PDCoV had significantly lower *N* mRNA (80% lower), N protein (90% lower) levels than in WT cells, and the virus titer was 2logTCID_50_ less (Fig. 6G through I). These results indicated that the downregulation of dynamin-2 strongly inhibits PDCoV entry into ST cells, further confirming that PDCoV entry depends on dynamin.

**Fig 7:**
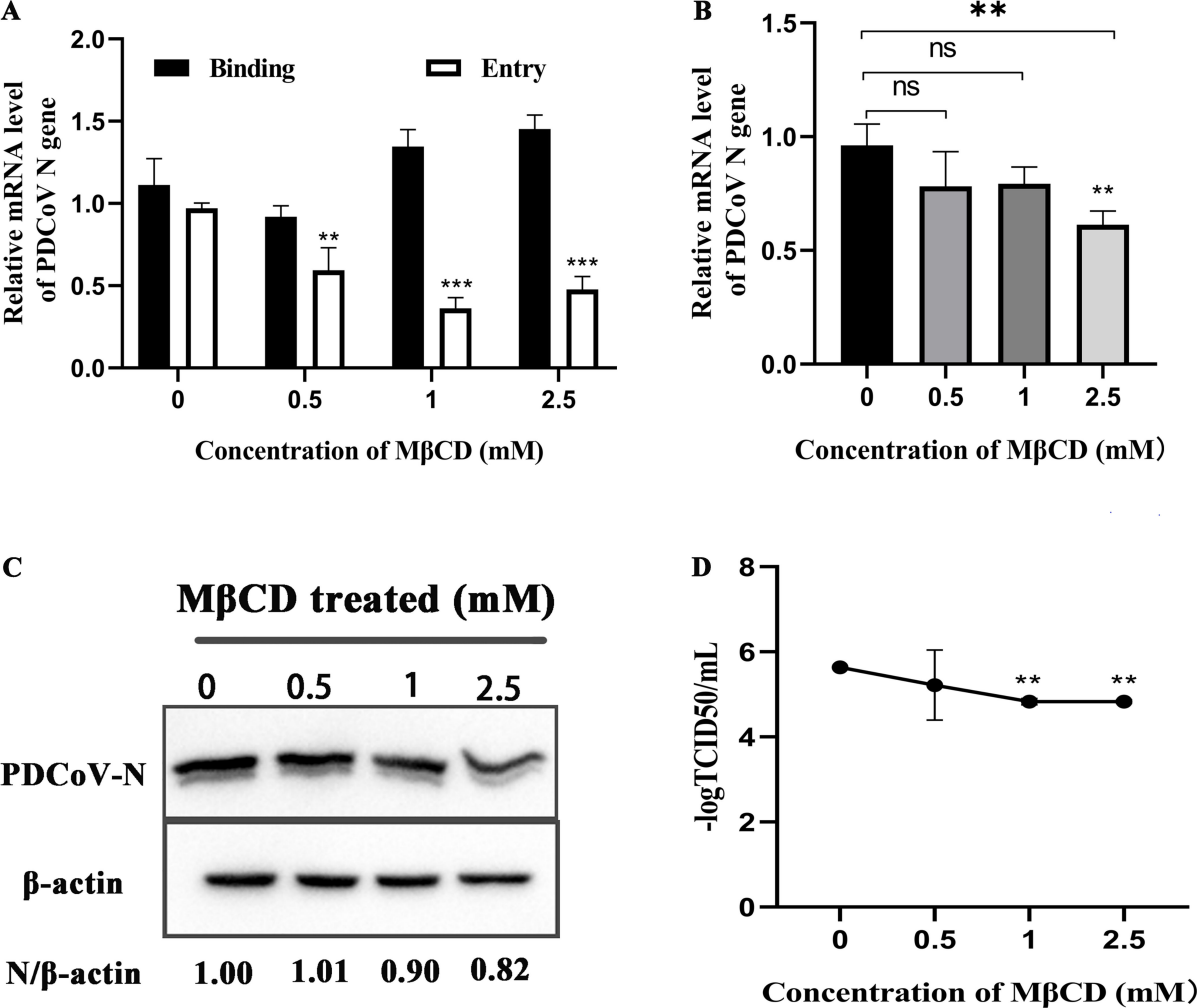
PDCoV entry into ST cells depends on cholesterol. (A) ST cells were pretreated with MβCD for 1 h at 37°C, then incubated with PDCoV (MOI = 5) for 1 h at 4°C (binding step), and then shifted to 37°C for 1 h (entry step). Viral *N* mRNA was quantitated by qRT-PCR. (B–D) Cells were mock-treated or treated with MβCD for 1 h followed by infection with PDCoV (MOI = 0.1) for 24 h. (B) *N* mRNA levels were quantitated by qRT-PCR. (C) Intracellular N protein levels were detected by Western blot. (D) Viral titers were determined by TCID_50_. ns: no significant difference.

Cholesterol is an important component of cell membranes and is essential for the formation of pits, it is also necessary for the entry of many coronaviruses into cells. To determine if PDCoV entry depends on cholesterol, ST cells were treated with MβCD (cholesterol extract) then incubated with PDCoV. Results showed that MβCD had no effect on PDCoV binding, but significantly inhibited entry (Fig. 7A). In ST cells treated with MβCD and infected with PDCoV for 24 h, *N* mRNA and protein levels were significantly decreased at 2.5 mM (the highest concentration tested). Virus titers were significantly lower in cells treated with 1 and 2.5 mM MβCD (Fig. 7B and C). These results demonstrate that MβCD inhibits PDCoV entry into ST cells, suggesting that PDCoV entry depends on cholesterol.

### Role of Rab proteins in PDCoV infection

**Fig 8:**
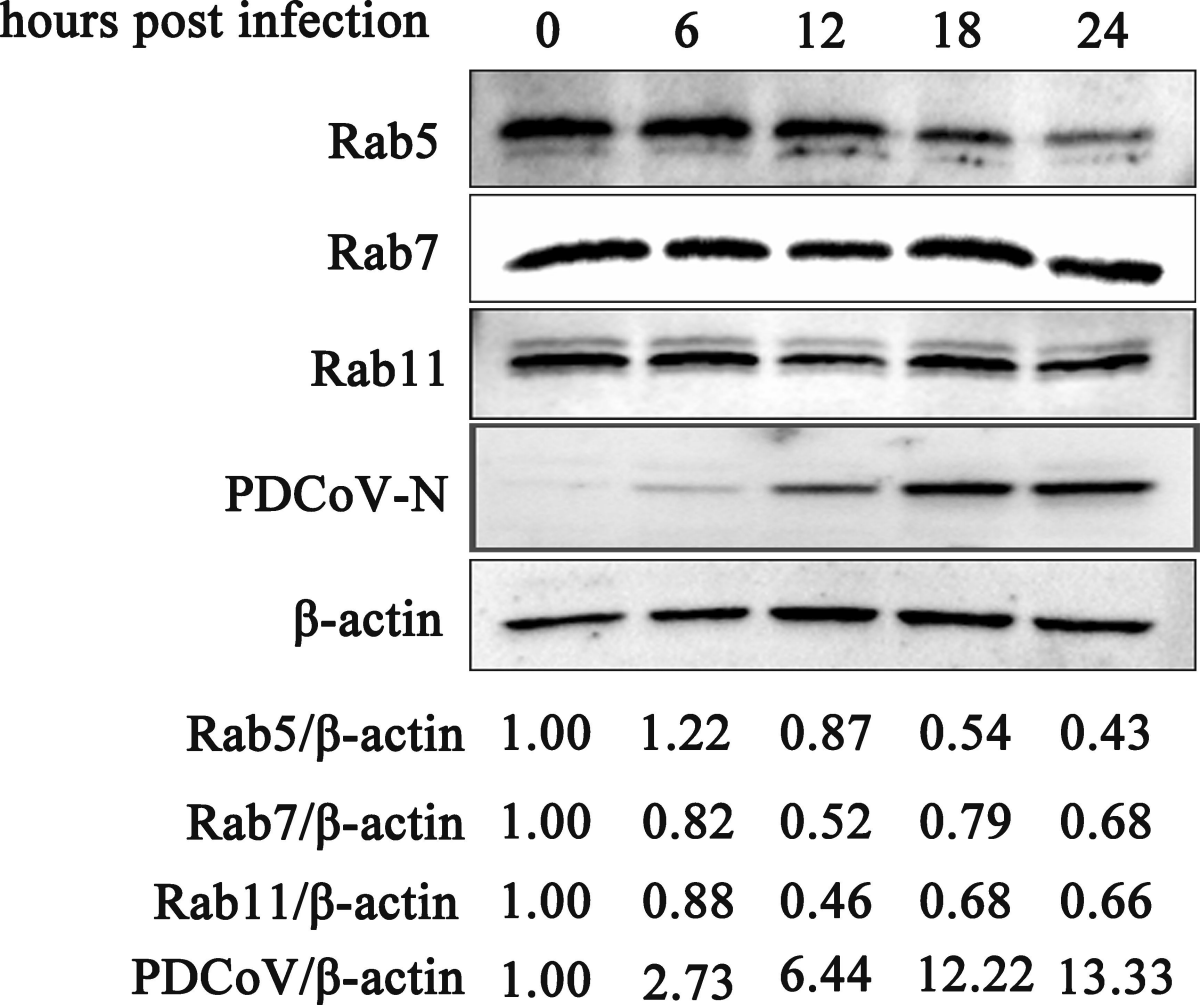
Levels of Rabs in PDCoV infected on ST cells. ST monolayers were incubated with PDCoV (MOI = 0.1) for 1.5 h at 37°C. Samples were collected for Western blotting analysis at the indicated times post-infection.

After entering the cell, the virus transports to specific compartments to complete membrane fusion and release the nucleocapsid for replication and maturation. However, how PDCoV transports in ST cells and which Rab proteins are involved remain unclear. To investigate PDCoV intracellular transport, we first determined the levels of Rab5, Rab7, and Rab11 in cells infected for 0, 6, 12, 18, and 24 h (Fig. 8). By quantitative analysis of gray scale of Western blots, we found that each of these Rabs decreased with increasing infection time, Rab7 and Rab11 decreased slightly, indicating that Rab7 and Rab11 may not be required for PDCoV infection.

**Fig 9:**
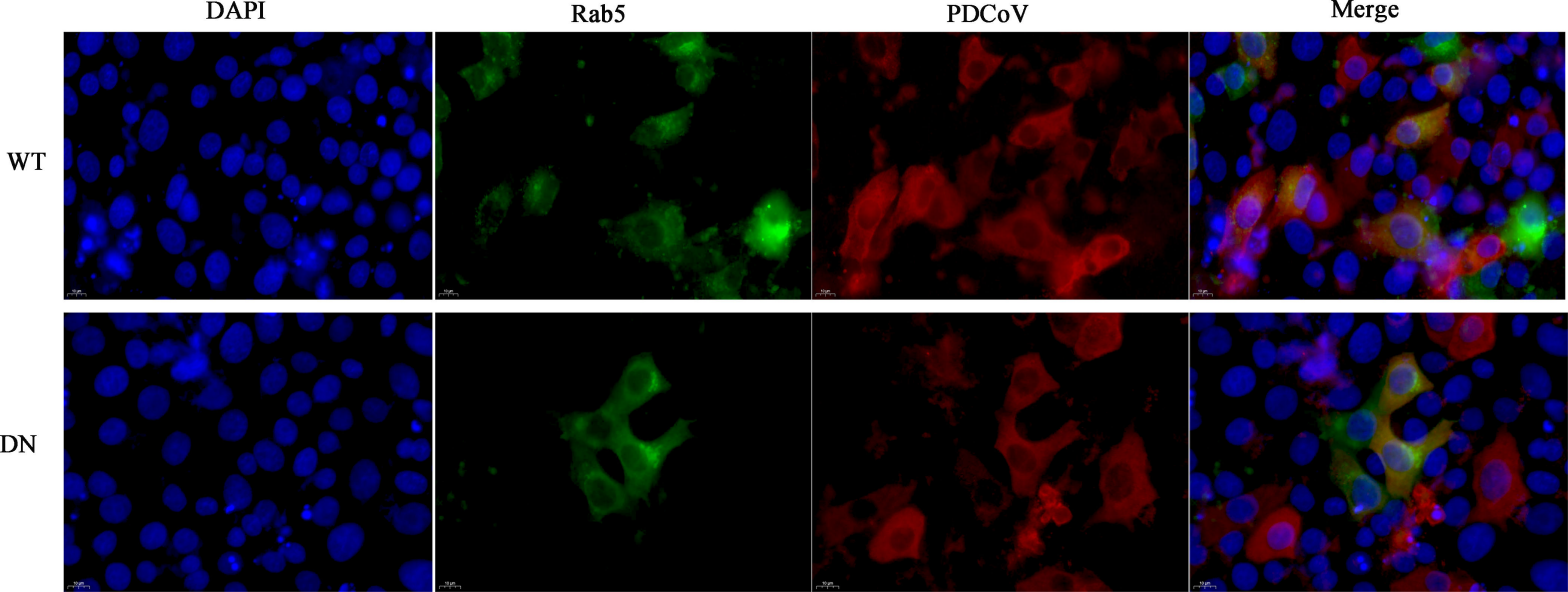
Effect of Rab5 WT overexpression and DN expression on PDCoV infection. ST cells were transfected with Rab5 WT or DN expressing plasmids for 24 h, followed by infection with PDCoV (MOI = 0.1) for 6 h. Cells were fixed and immuno-stained. The magnification is 80×.

**Fig 10:**
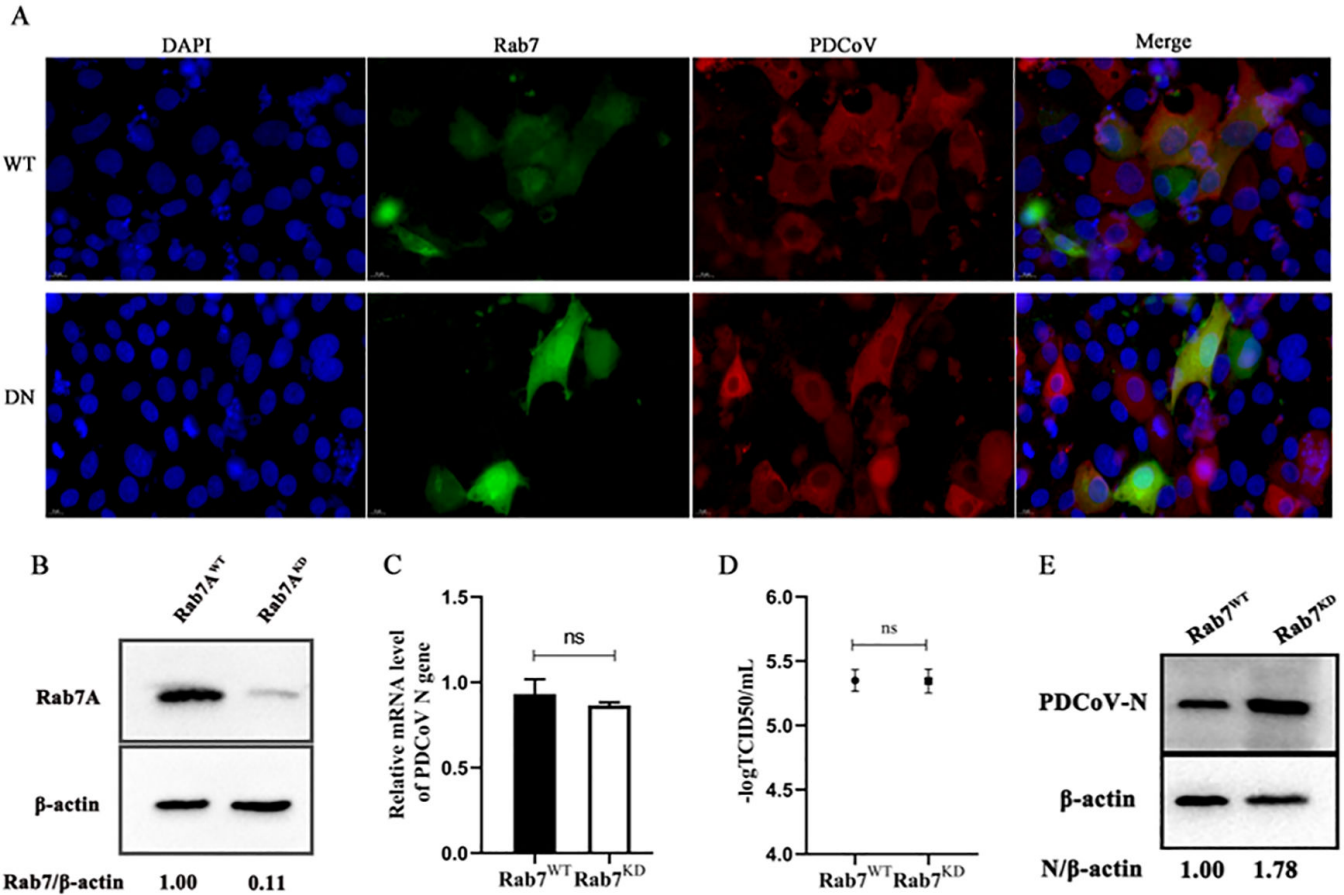
Effect of Rab7 on PDCoV infection. (A) ST cells were transfected with Rab7 WT or DN expressing plasmids for 24 h, followed by infection with PDCoV (MOI = 0.1) for 6 h. Cells were fixed and immuno-stained. The magnification is 80×. (B) Knockdown efficiency was verified by Western blotting. (C–E) Rab7 knockdown and normal cells were infected with PDCoV (MOI = 0.1) for 24 h. *N* mRNA levels, viral titer, and N protein levels were determined by (C) qRT-PCR, (D) TCID_50_, and (E) Western blotting. ns: no significant difference.

**Fig 11:**
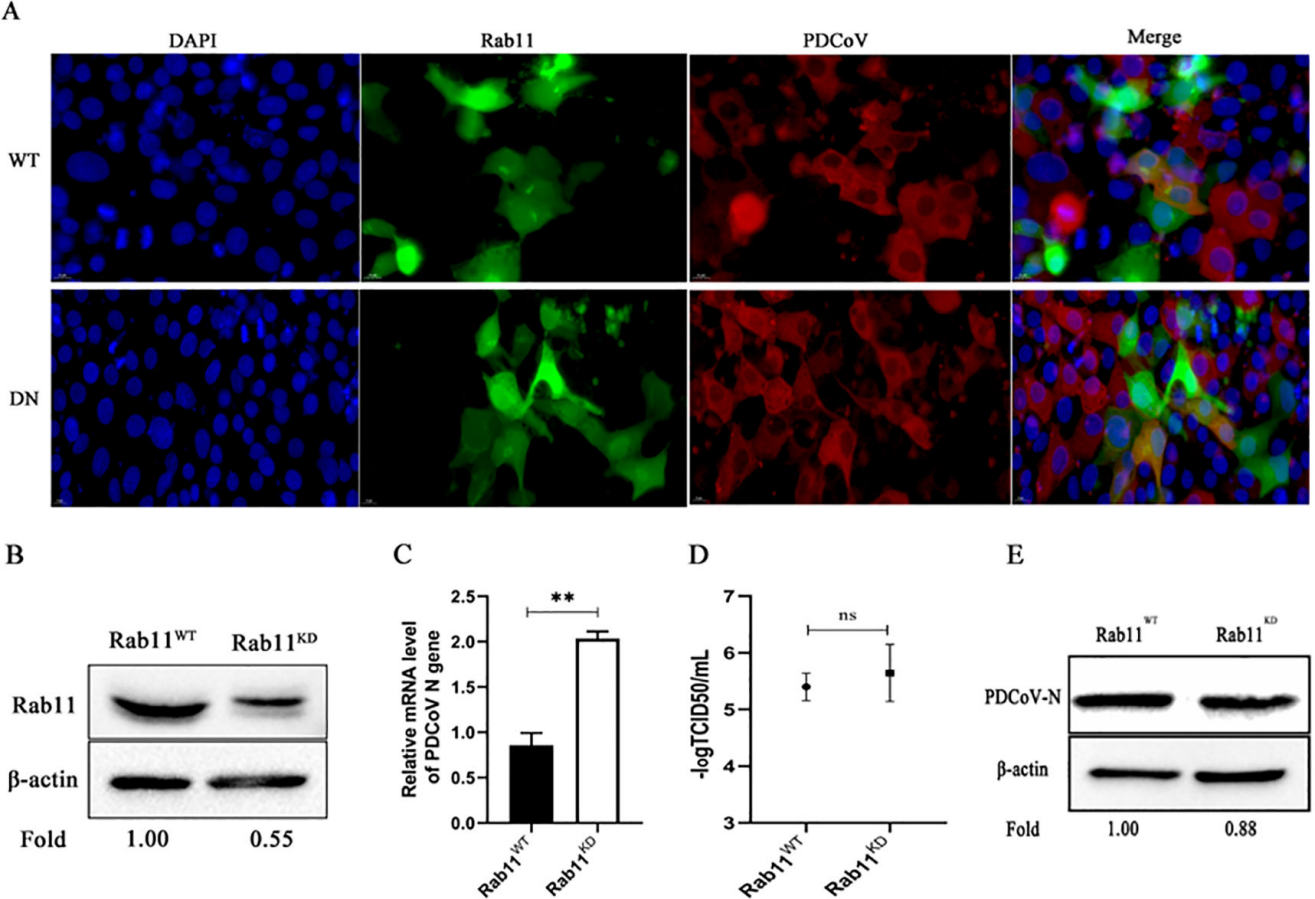
Effect of Rab11 on PDCoV infection. (A) ST cells were transfected with WT or DN expressing Rab11 expressing plasmids for 24 h, followed by infection with PDCoV (MOI = 0.1) for 6 h. Cells were fixed and immuno-stained. The magnification is 80×. (B) Knockdown efficiency was verified by Western blotting. (C–E) Rab11 knockdown and normal cells were infected with PDCoV (MOI = 0.1) for 24 h. *N* mRNA levels, viral titer, and N protein levels were determined by (C) qRT-PCR, (D) TCID_50_, and (E) Western blotting. *P < 0.05, **P < 0.01, ***P < 0.001, and ns: no significant difference.

Subsequently, plasmids expressing WT and DN Rab5, Rab7, and Rab11 were separately transfected into ST cells followed by infection with PDCoV. By indirect immunofluorescence we found that PDCoV and each Rab colocalized in WT^+^ and DN^+^ cells (Fig. 9A, 10A, and 11A). The role of Rabs 7 and 11 was further assessed by constructing knockdown cells (we failed to construct knockdown cells of Rab5). As can be seen from Fig. 10B and 11B, respectively, Rab7 and Rab11 were significantly knocked down compared to normal cells. Rab7 knockdown cells infected with PDCoV had no significant difference in *N* mRNA levels or titer compared to normal cells, although N protein levels were significantly higher (Fig. 10C through E). Rab11 knockdown cells infected with PDCoV had significantly greater *N* mRNA levels, although lower N protein levels, compared to normal cells; there was no significant difference in titer (Fig. 11C through E). Taken together, these results show that PDCoV entry into ST cells does not require Rab5, Rab7, or Rab11.

## DISCUSSION

Endocytosis is an effective mechanism used by many viruses to overcome the physical barrier of the cytoplasmic membrane and enter the cells to initiate productive infection. The endocytosis pathways depend on the activation of specific cellular signaling pathways driven by virus-cell interactions. Understanding the signaling pathways and the related mechanisms that trigger viral entry is central to understanding host-cell interactions and viral pathogenesis. Although previous studies have clarified the endocytosis mechanisms used by PDCoV in some cells (18 – 20), PDCoV infects many organs (heart, liver, spleen, lung, and kidney) other than its main target organ, the intestine, and *in vitro*, PDCoV infects numerous types of cells (ST, PK-15, LLC-PK, CEF, DF-1, and others) (1, 39). Given the broad host range of PDCoV and its ability to spread quickly, understanding the differences in entry strategies used by the virus is worth further study.

### Chemical inhibition of endocytosis

The use of chemical inhibitors is a classic method to study viral entry and has been widely used in many studies. For example, Liang et al. (40) treated ST cells with CPZ and dynasore and found that the efficiency of CSFV internalization was significantly reduced. Nanbo et al. used CytoD, wortmannin, and EIPA to inhibit the macropinocytosis pathway and found that ebolavirus entry was significantly inhibited (29). Li et al. used MβCD and NH_4_Cl to treat DF-1 and Vero cells and found that an acidic environment and cholesterol were important for effective infection of Muscovy duck reovirus (41). In this study, we used CPZ, nystatin, and EIPA to block the CME, CavME, and macropinocytosis pathways, respectively. And found that CPZ effectively inhibited PDCoV infection, indicating that PDCoV entry into ST cells may be via the CME pathway. Using dynasore, MβCD, and NH_4_Cl we found that PDCoV entry requires dynamin and cholesterol.

Many coronaviruses need cholesterol on the viral capsule or cell membrane to enter cells (17, 42), and some rely on an acidic environment to enter the cells, such as IBV and SARS (21, 43). PDCoV can enter cells through endocytosis and the cell membrane surface pathway, so it is not sufficient to inhibit PDCoV entry using nucleosome inhibitors alone (18, 44). Here, in ST cells, PDCoV infection declined only at higher concentrations of MβCD (Fig. 7) and increased after using NH_4_Cl (Fig. 5). These results are consistent with our results in PK-15 cells (20). We speculate that low concentrations of MβCD do not adequately antagonize cholesterol in the cell membrane and viral capsule, and that although the endocytosis pathway is inhibited by NH_4_Cl, NH_4_Cl provides favorable conditions for PDCoV entry via the cell membrane surface pathway.

### Interference of PDCoV endocytosis using dominant-negative mutants

Use of dominant-negative mutants is an effective approach for studying the function of the genes and proteins. Expression of dominant negative mutants not only disrupts normal biological function of a given protein but also competes with its wild-type version, thereby producing an inhibited or blocked regulatory effect (45, 46). To further investigate PDCoV entry via the CME pathway, we used dominant-negative mutants of EPS15 and dynamin-2, both important regulatory proteins of the CME pathway. We also used a dominant-negative mutant of caveolin-1, which is important in the CavME pathway. Cells expressing DN EPS15 or dynamin-2 were not well infected by PDCoV (Fig. 2E and 6E), while cells expressing DN caveolin-1 were equally well infected and those expressing WT caveolin-1 (Fig. 3E). These results showed that interference with the normal functions of EPS15 and dynamin-2 effectively inhibits PDCoV internalization, further demonstrating that PDCoV entry into ST cells is via the CME pathway and depends on dynamin.

Using CRISPR/Cas9 and lentiviruses to construct cells deficient in clathrin heavy chain, caveolin-1, and dynamin-2, we found that PDCoV infection decreased significantly in CLTC and DNM2 knockdown cells, further demonstrating that PDCoV entry depends on the CME pathway and requires dynamin. As measured by *N* mRNA levels, PDCoV infection in CAV1 knockdown cells increased significantly, although viral titers remained equivalent to those in WT cells, and N protein levels decreased.

### PDCoV utilizes clathrin-mediated endocytosis for entry into ST cells

Our results revealed that PDCoV strain CHN-SC-2015 enters ST cells through clathrin-mediated endocytosis while Fang et al. found that PDCoV strain CHN-HN-2014 enters ST cells through caveolae-mediated endocytosis (19). Due to the multidirectional regulation of endocytosis and cell-type dependence, PDCoV may enter cells via more than one pathway, and there are clearly different entry strategies between virus strains (38, 47). Bao et al. have shown that influenza A viruses (IAVs) enter host cells via extracellular Ca^2+^ influx-involved clathrin- and dynamin-dependent endocytosis (48). PDCoV infection increased intracellular Ca^2+^ levels in IPI-2I cells, and treatment with Ca^2+^ chelators and channel blockers significantly decreased viral yield (49). It remains for further study whether PDCoV utilizes the CME pathway by regulating Ca^2+^ influx like IAVs.

Our previous study showed that PDCoV (CHN-SC-2015) entry into PK-15 cells requires dynamin and cholesterol (20). Fang et al. also found that PDCoV entry into IPI-2I cells required dynamin and cholesterol (19). Here, we found that PDCoV entry into ST cells also depends on dynamin and cholesterol. As a GTPase, dynamin plays an important role in both the CME and CavME pathways. The formation of endocytic vesicles in these pathways requires the "shear" effect of dynamin to separate vesicles from the cell membrane.

Cholesterol is a ubiquitous component of the cytoplasmic membrane, and most viral capsules contain cholesterol (42, 50). Many coronaviruses require cholesterol to enter host cells and PDCoV is no exception (17). Since the entry of PDCoV CHN-SC-2015 and CHN-HN-2014 requires dynamin and cholesterol, we speculate that this may be the common point of PDCoV entry and can be used as a target for antiviral therapies.

### Role of Rab proteins in PDCoV infection

Rab5 is a marker of early endosomes, Rab7 is a marker of late endosomes, and Rab11 is a marker of circulating endosomes (51, 52). Fang et al. showed that transport of PDCoV requires Rab5 and Rab7 to enter IPI-2I cells (19). Wang et al. found that early and late endosomes are required for intracellular transport of IBV through the co-localization of R18 labeled virus with transport vesicle markers (Rab5 and Rab7) (24). Li et al. found that Rab5- and Rab7-dependent pathways were required for the initiation of PHEV productive infection, and endogenous Rab5 was crucial for the viral progression (25). It is therefore of interest to explore the transport of PDCoV (CHN-SC2015) in ST cells. Here, we found that neither PDCoV entry nor replication in ST cells requires Rab7 or Rab11. We attempted to interfere with the expression of Rab5 using siRNA and lentivirus packaging technology but failed. However, we did demonstrate that PDCoV entry does not require Rab5.

The entry of HSV-1 into cells does not depend on Rab5, Rab7, and Rab11. Endocytic entry of HSV-1 is independent of the canonical lysosome-terminal pathway, and a nontraditional endocytic route may be employed, such as one that intersects with the trans-Golgi network (53). Therefore, we suspect that the entry of PDCoV into ST cells may be similar to that of HSV-1, and its transport in ST cells does not require the participation of traditional endosome system.

In conclusion, our data show that PDCoV entry into ST cells occurs through clathrin-mediated endocytosis and is dependent on dynamin and cholesterol. PDCoV entry into ST cells does not depend on caveolae-mediated endocytosis, macropinocytosis, and a low pH environment. PDCoV entry and transport do not require Rab5, Rab7, or Rab11. Our findings contribute to the knowledge base of PDCoV cellular entry mechanisms, providing possible targets for antiviral drug development.
